# A pharmacokinetic binding model for bevacizumab and VEGF_165_ in colorectal cancer patients

**DOI:** 10.1007/s00280-015-2701-3

**Published:** 2015-02-17

**Authors:** Eirini Panoilia, Emilie Schindler, Epaminontas Samantas, Gerasimos Aravantinos, Haralabos P. Kalofonos, Christos Christodoulou, George P. Patrinos, Lena E. Friberg, Gregory Sivolapenko

**Affiliations:** 1 Department of Pharmacy, University of Patras, Rio-Patras, Greece; 2 Department of Pharmaceutical Biosciences, Uppsala University, Uppsala, Sweden; 3 3rd Department of Medical Oncology, “Agii Anargiri” Cancer Hospital, Kalyftaki-Nea Kifissia, Greece; 4 2nd Department of Medical Oncology, “Agii Anargiri” Cancer Hospital, Kalyftaki-Nea Kifissia, Greece; 5 Division of Medical Oncology, University Hospital of Patras, Rio-Patras, Greece; 6 2nd Department of Medical Oncology, “Metropolitan” Hospital, Athens, Greece

**Keywords:** Bevacizumab, VEGF, Population modeling, VEGF polymorphisms, Colorectal cancer

## Abstract

**Purpose:**

To characterize the population pharmacokinetics of bevacizumab, its binding properties to VEGF_165_ and the effect of demographic data and VEGF-A polymorphisms on the interplay between bevacizumab serum pharmacokinetics and VEGF_165_ serum concentrations in patients with colorectal cancer stage IV.

**Methods:**

Bevacizumab and VEGF_165_ data were collected from 19 adult patients with metastatic colorectal cancer enrolled in an observational clinical study. Bevacizumab was administered with one of the following combinations: 5-FU/Leucovorin/Irinotecan, 5-FU/Leucovorin/Oxaliplatin, Capecitabine/Irinotecan at doses ranging from 5 to 10 mg/kg every 2 or 3 weeks. Data analysis was performed using nonlinear mixed-effects modeling implemented in NONMEM 7.3.

**Results:**

A target-mediated drug disposition model adequately described bevacizumab concentration changes over time and its binding characteristics to VEGF_165_. The estimated clearance of bevacizumab was 0.18 L/day, the free VEGF_165_ levels at baseline were 212 ng/L, and the elimination rate constant of free VEGF_165_ was 0.401 day^−1^. Body weight was allometrically included in all PK parameters.

**Conclusion:**

The final model adequately described the pre- and post-dose concentrations of total bevacizumab and free VEGF_165_ in patients with colorectal cancer. Model parameters were consistent with those previously reported for patients with solid tumors. Correlations between the binding affinity of bevacizumab and the VEGF-2578C/A and VEGF-634G/C polymorphisms were noticed.

**Electronic supplementary material:**

The online version of this article (doi:10.1007/s00280-015-2701-3) contains supplementary material, which is available to authorized users.

## Introduction

Bevacizumab, the active substance of Avastin, is a recombinant humanized IgG1 monoclonal antibody with multiple cancer indications. It has been approved in combination with chemotherapy for the treatment of metastatic colorectal cancer (CRC), metastatic breast cancer, unresectable advanced, metastatic or recurrent non-small cell lung cancer, advanced or metastatic renal cell cancer, advanced epithelial cancer of the ovary, the fallopian tube or the peritoneum and as a single agent for advanced glioblastoma [1–3].

Bevacizumab targets circulating vascular endothelial growth factor (VEGF or else VEGF-A), a vasculogenesis and angiogenesis regulator that is overexpressed in most human tumors. By blocking VEGF-A binding to its receptors (VEGFR-1 and VEGFR-2) on the surface of endothelial cells, bevacizumab inhibits tumor angiogenesis, growth and metastases [1, 3–6].

There are five major isoforms of the human VEGF-A glycoprotein (VEGF_121_, VEGF_145_, VEGF_165_, VEGF_189_, VEGF_206_) produced by the alternative splicing of the VEGF-A gene. All isoforms differ in the amino acid length and the binding ability to heparin and heparin sulfate proteoglycans found on the cell surface or in the extracellular matrix. VEGF_145_, VEGF_189_ and VEGF_206_ are tightly bound to the cell surface and the extracellular matrix, VEGF_121_ is freely diffusible, and VEGF_165_ is moderately diffusible. VEGF_165_ is the most abundant and potent stimulator of angiogenesis compared to the other isoforms. It is overexpressed in several cancer types and is related to the ability of a tumor to grow, invade and spread [7, 8].

Several clinical trials in CRC patients indicate an inverse relationship between the concentration of VEGF-A in serum or tumor tissue and the clinical outcome [9–11]. Furthermore, certain VEGF-A polymorphisms have been associated with altered VEGF-A production or promoter activity, causing variability in response to treatment [12–14]. The VEGF-2578CC, VEGF-1154GG and VEGF-634CC genotypes have been correlated with higher VEGF-A production than other genotypes for VEGF-2578C/A, VEGF-1154G/A and VEGF-634G/C polymorphisms [13, 15]. Thus, free serum VEGF-A levels could serve as a useful biomarker for reflecting the anti-angiogenic activity of bevacizumab and for indicating when dose adjustments are needed to achieve sufficient VEGF-A blockade.

Monoclonal antibodies exhibit more complex concentration–time [pharmacokinetic (PK)] and effect–concentration [pharmacodynamic (PD)] characteristics than small molecules. The main elimination pathway is proteolytic catabolism throughout the body (linear, nonspecific clearance) and not hepatic metabolism or renal excretion. However, the disposition of monoclonal antibodies is often influenced by their high affinity binding to their molecular targets and subsequent degradation of the monoclonal antibody–target complexes via fluid phase or receptor-mediated endocytosis. This phenomenon is known as target-mediated drug disposition (TMDD), and it usually leads to nonlinear, saturable distribution and elimination [16, 17]. In recent years, population PKPD modeling has proven to be a useful tool to elucidate the dose–response relationships, to explain inter-individual variability in the observed drug exposure or response and to guide dose selection to achieve the optimal benefit–risk ratio for a given anticancer treatment [18–20].

TMDD modeling has been generally used to describe the dynamics of the drug–target interaction, as it can provide valuable information on the mechanism of the underlying PKPD relationship [17, 21, 22]. The full TMDD model is a complex system of differential equations describing the concentrations of the free drug, the free target and the drug–target complex. Model parameters may not be identifiable when limited experimental data on target, total and free drug concentrations are available. In that case, simplifications of the full TMDD model with fewer parameters can be applied such as the quasi-equilibrium, quasi-steady-state (QSS) and Michaelis–Menten approximation [23, 24].

The full TMDD model is based on the following assumptions: (1) the drug–target binding is a simple, one-to-one binding process, (2) the drug can only bind to its specific molecular target, (3) the drug–target binding takes place in the central and not in the peripheral or depot compartments, (4) only the free drug can distribute into the peripheral compartment, (5) the drug–target complex is totally eliminated, and (6) the free target production and degradation rates are constant and independent of the drug or target concentrations. The main assumption of the QSS approximation, which differentiates it from the full TMDD model or other TMDD approximations, is that the change in the drug–target complex concentration is negligible on the time scale of the other system processes. The free drug, the free target and the drug–target complex are assumed to be in a QSS. The QSS approximation is usually preferred when only total drug and target concentrations are available and is applied for drugs with fast binding, dissociation and internalization rates [22–24].

The aim of the present study was to describe the PK of bevacizumab, its binding properties to VEGF_165_ and the effect of individual patient characteristics on the relationship between bevacizumab and VEGF_165_ in patients with stage IV CRC using a population modeling approach. To the best of our knowledge, a limited number of population PK studies for bevacizumab have been conducted so far [25, 26]. Moreover, little quantitative pharmacology research has been performed to establish the impact of VEGF-A polymorphisms and other relevant covariates on treatment response [27–29]. Therefore, it is anticipated that the development of a binding model for bevacizumab in patients with metastatic CRC would yield a better mechanistic understanding of the biological system, as it could characterize the in vivo interaction of the drug with its soluble target, VEGF_165_. It would further elucidate the PK characteristics of the drug, its effect on the free target time course and provide more information on target affinity and the influence of covariates (e.g., genetics).

## Materials and methods

### Patients and study design

Nineteen subjects were enrolled in this observational (non interventional) study. Eligible patients had CRC stage IV documented by histology (biopsy through colonoscopy or surgery), CT scans (thoracic, abdominal and brain scans), bone scintiscans and the measurement of cancer biomarkers (CEA and CA 19.9); Eastern Cooperative Oncology Group (ECOG) performance status ≤2; no chemotherapy or radiotherapy within 3 months of first study treatment; were at least 18 years of age. Prior to enrollment, all patients had undergone a complete history and physical examination. Standard hematologic and biochemical laboratory tests had also been conducted to assess an adequate bone marrow, liver and renal function as defined by: white blood cells count (WBC) ≥ 2500/μL, absolute neutrophil count (ANC) ≥ 1500/μL, platelets ≥ 75,000/μL, serum creatinine ≤ 1.5 × upper limit of normal (ULN), urine protein ≤ 2 g/24 h, total serum bilirubin ≤ 3 mg/dL, AST (SGOT)/ALT (SGPT) ≤ 2 × ULN. The research was conducted in accordance with the Declaration of Helsinki, and all the appropriate approvals were obtained by the relevant Ethics Committees. Signed informed consent was provided by all participants before the initiation of the study.

The current study was carried out in three Greek oncology clinics, at the Department of Medical Oncology of Agii Anargiri Cancer Hospital, at the Department of Medical Oncology of Metropolitan Hospital in Athens and at the Division of Oncology of Patras University Hospital in Rio.

Bevacizumab (Avastin^®^, Roche Registration Ltd.) was administered as an intravenous infusion at a dose of 5 mg/kg, in combination with 5-Fluorouracil/Leucovorin/Irinotecan (BEV-FOLFIRI treatment) or 5-Fluorouracil/Leucovorin/Oxaliplatin (BEV-FOLFOX treatment), in 2-week cycles or at a dose of 7.5 mg/kg together with Capecitabine/Irinotecan (BEV-CAPIRI treatment) in 3-week cycles. One patient received the BEV-FOLFIRI treatment at a dose of 10 mg/kg in 2-week cycles. The duration of first infusion was 90 min. If no infusion-related symptoms were observed, subsequent infusions were given over 60 or 30 min.

Subjects on BEV–FOLFIRI or BEV–FOLFOX treatment received initially six cycles of bevacizumab and those who responded to treatment, as defined by Response Evaluation Criteria in Solid Tumors [30], continued with the same treatment for other six cycles. The responders, after the completion of 12 cycles in total, continued with bevacizumab monotherapy till disease progression. In that case, they were administered bevacizumab at a dose of 5 mg/kg every 2 weeks.

Subjects on BEV–CAPIRI treatment received initially three or four cycles of bevacizumab (the number of cycles depends on the lines of treatment the patients had undergone before the initiation of the current treatment). After the completion of eight or nine cycles, the responders continued either with the same treatment or bevacizumab monotherapy upon development of disease progression at the recommended dose (7.5 mg/kg every 3 weeks).

Pre- and post-dose (after the end of infusion) concentrations of total bevacizumab (free and bound to one molecule of VEGF_165_) and free VEGF_165_ (unbound to bevacizumab) were measured in serum during several cycles of treatment. Two blood samples (one pre-dose and one post-dose) were drawn on day 1 of the following cycles: (1) 3, 6, 8, 12, 18 and 24 (patients on BEV–FOLFIRI or BEV–FOLFOX treatment), (2) 2, 4, 5, 8 and 11 (patients on BEV–CAPIRI treatment). One pre-dose blood sample was also collected on day 1 of cycle 1, intended only for free VEGF_165_ analysis. The sample collection schedule for total bevacizumab and free VEGF_165_ measurements is shown in Supplemental Fig. 1. The genotypes for VEGF-2578C/A, VEGF-1154G/A and VEGF-634G/C single-nucleotide polymorphisms (SNPs) were also determined in blood.

### Measurement of total bevacizumab in serum

Blood samples were collected in serum separator tubes and were allowed to clot for 30 min. After centrifugation at 1000×*g* for 20 min, the serum was removed and stored in aliquots at ≤−20 °C until analysis.

The concentration of total (free and bound to one molecule of VEGF_165_) bevacizumab in serum was measured using a previously published enzyme-linked immunosorbent assay (ELISA), where the detection limit was 0.033 mg/L and the range of linearity was between 5 and 75 mg/L with precision 5.6 % [expressed as coefficient of variation (CV) percentage]. Standards of 0.24, 0.47, 0.94, 1.88, 3.75, 7.5, 15 and 30 mg/L were used to generate the standard curve, which are well above the detection limit of the assay and within the range of linearity [31].

Microtiter Nunc Maxisorp 96-well plates were coated with recombinant human VEGF_165_ (R&D Systems^®^ Europe) at a concentration of 0.15 mg/L in carbonate–bicarbonate buffer (1 M, pH 9.6) overnight at 4 °C (100 μL/well). After washing four times with phosphate-buffered saline (PBS) containing 0.05 % Tween 20, the wells were blocked with PBS containing 1 % BSA (200 μL/well) and were incubated for 2 h at room temperature. Afterward, the plates were washed and 100 μL of 1:100 diluted standards and samples in 1 % PBS–BSA was added and were incubated for 1 h at 37 °C in an incubator shaker. Then, the plates were washed again, and 100 μL of peroxidase-conjugated goat antihuman IgG specific for Fc fragment (AbD Serotec^®^, A Bio-Rad Company) diluted in 1 % PBS–BSA was added to each well. After 1-h incubation at room temperature followed by washing, 100 μL OPD (Sigma-Aldrich) was added and the reaction was allowed to develop at room temperature in the dark. The color reaction was stopped with the addition of sulfuric acid (2 M, 50 μL/well).

The optical density was measured at 450 nm with a correction at 650 nm using an ELISA plate reader (ThermoMax, Molecular Devices). Duplicate readings for 1:100 diluted standards and samples were performed.

The best fit line of the standard curve was determined by regression analysis using OriginPro 8.0 software (OriginLab^®^ Corporation). The concentrations read from the standard curve were multiplied by the dilution factor.

### Measurement of free VEGF_165_ in serum

Blood samples were collected in serum separator tubes and were allowed to clot for 30 min. After centrifugation at 1000×*g* for 20 min, the serum was removed and stored in aliquots at ≤−20 °C until analysis.

The concentration of free VEGF_165_ (unbound to bevacizumab) in serum was measured by a commercially available ELISA kit for VEGF_165_ (Quantikine^®^ human VEGF, R&D Systems^®^ Europe). The detection limit of the assay was 9 ng/L, and the precision was 6.7 % (CV %) [32]. According to the manufacturer, this ELISA assay has not been tested yet for interference with the detection of free or total (free and bound to bevacizumab) VEGF_165_ in the presence of bevacizumab. To confirm the hypothesis that it can only discriminate and quantitate free VEGF_165_, we measured VEGF_165_ concentrations in samples after the addition of increasing concentrations of bevacizumab. VEGF_165_ standards (1000 and 250 ng/L, respectively) were mixed with increasing VEGF_165_-to-bevacizumab molar ratios of 1:0, 1:0.1, 1:1 and 1:1000.

The assay procedure is briefly described below. Plates pre-coated with a mouse anti-VEGF antibody were used to capture VEGF_165_ in standards or samples. Any unbound proteins were washed off and a peroxidase-conjugated polyclonal antibody specific for VEGF_165_ was added. Then, the plates were washed again and tetramethylbenzidine substrate solution was added. A blue color was developed in proportion to the amount of VEGF_165_ present in the ELISA samples. Color development was stopped with the addition of sulfuric acid.

The optical density was measured at 450 nm with a correction at 550 nm using an ELISA plate reader (ELx800™, BioTek Instruments). All standards’ and samples’ readings were performed in duplicate.

A standard curve was generated with VEGF_165_ concentrations ranging from 31.2 to 2000 ng/L. The best fit line was determined by regression analysis using OriginPro 8.0 software (OriginLab^®^ Corporation).

### VEGF genotyping

Genomic DNA was isolated from blood (3 mL) using the Gentra Puregene Blood kit (QIAGEN). DNA concentrations were determined by measuring the optical density at 260 nm with a UV–Vis spectrophotometer (NanoDrop 2000, Thermo Fisher Scientific). DNA purity, which is indicated by the ratio of optical density at 260 and 280 nm, was 1.7–1.9.

VEGF polymorphisms (−2578C/A (rs699947), −1154G/A (rs1570360) and −634G/C (rs2010963) on VEGF genomic DNA) were analyzed by polymerase chain reaction (PCR) using the KAPA2G Fast HotStart ReadyMix kit (Kapa Biosystems, fidelity of the DNA polymerase: 1 error per 1.7 × 10^5^ nucleotides incorporated). The primers VEGF-1154 For 5′-TTCAGGCTGTGAACCTTGG-3′, VEGF-1154 Rev 5′-GGGCGGTGTCTGTCTGTC-3′, VEGF-634 For 5′-TTCAGGCTGTGAACCTTGG-3′, VEGF-634 Rev 5′-GGGCGGTGTCTGTCTGTC-3′, VEGF-2578 For 5′-AGCAACATGTGCTGAGGATG-3′, VEGF-2578 Rev 5′-CCCTTTTCCTCCAACTCTCC-3′ were used to amplify fragments of the VEGF gene.

PCR was done with 40 cycles at 95 °C for 2 min, at 60–65 °C (VEGF-1154G/A and VEGF-634G/C) or 63 °C (VEGF-2578C/A) for 15 s and at 72 °C for 1 s. The reaction was preceded by a primary denaturation step at 95 °C for 15 s.

PCR products were separated on 1 % w/v agarose gels stained with ethidium bromide and were purified using the PCR and DNA Fragment Purification kit (Dongsheng Biotech, DNA purity 1.7–1.9). Sanger DNA sequencing by capillary electrophoresis was applied to detect the genotypes for VEGF-2578C/A (rs699947), VEGF-1154G/A (rs1570360) and VEGF-634G/C (rs2010963) SNPs (3130 genetic analyzer, Applied Biosystems^®^) [33, 34].

### Model development

The nonlinear mixed-effects modeling software NONMEM^®^ 7.3 (Icon Development Solutions, Ellicott City, MD, USA) was used in the data analysis. All population parameter estimates were obtained with the first-order conditional estimation method with interaction. The graphical representation of the data and model diagnostics was performed with the software tool Xpose (version 4.3.5) [35]. The PsN toolkit (version 4.2.0) was used for the implementation of computer-intensive statistical methods (stepwise covariate model building and randomization test) [36]. Typical concentration–time profiles for total bevacizumab, total and free VEGF_165_ were generated using Berkeley Madonna (version 8.3.18, Kagi Shareware, Berkeley, CA, USA).

Inter-individual variability in model parameters was tested assuming a log-normal distribution described by an exponential model (Eq. 1),

1 $$P_{i} = P_{p} \cdot \exp \left( {\eta_{i,p} } \right)$$

where *P*_*i*_ represents the parameter estimate for the *i*th individual, *P*_*p*_ the typical parameter estimate in the population and *η*_*i,p*_ the random variable for the *i*th individual from a normal distribution with a mean of zero and an estimated variance of *ω* ^2^. Proportional, additive and combined error models on normal scale or additive and combined error models on log-transformed scale were explored to describe the unexplained residual variability. The magnitude of inter-individual and residual variability was expressed as CV %.

The likelihood ratio test was used to assess whether the difference in the objective function (ΔOFV) between different (sub)models (assumed to be *χ* ^2^ distributed) was statistically significant. The evaluation of basic goodness-of-fit plots, shrinkage [37] and parameter uncertainty were also taken into account for model discrimination.

Plots of individual empirical Bayes (post hoc) estimates of the PK and VEGF-related parameters versus covariates were explored for the identification of potential parameter–covariate relationships. The choice of the covariate model was based on a stepwise covariate model procedure, a PsN feature that implements forward selection and backward elimination of covariates to a model according to statistical criteria [36]. A decrease in OFV of 3.84 points for forward inclusion (*p* < 0.05, for one parameter difference) and an increase of 6.64 points for backward deletion (*p* < 0.01, for one parameter difference) were considered significant.

The following covariates were tested for significance on model parameters: actual body weight, age, gender and VEGF-A SNPs (−2578C/A, −1154G/A and −634G/C). Linear and power parameterizations were considered for the continuous covariates (Eqs. 2, 3, respectively), whereas the categorical covariates were tested in linear equations (Eq. 4). The equations are shown below:

2 $$P_{i} = P_{p} \cdot \left[ {1 + \theta \cdot \left( {{\text{Cov}}{-}{\text{Cov}}_{{{\text{med}} .}} } \right)} \right] \cdot \exp \left( {\eta_{i,p} } \right)$$

3 $$P_{i} = P_{p} \cdot \left( {\frac{\text{Cov}}{{{\text{Cov}}_{{{\text{med}} .}} }}} \right)^{k} \cdot \exp \left( {\eta_{i,p} } \right)$$

4 $$P_{i} = P_{p} \cdot \left( {1 + \theta \cdot {\text{Cov}}} \right) \cdot \exp \left( {\eta_{i,p} } \right)$$

In these equations, Cov represents the covariate and Cov_med._ the median value of the covariate in the study population. *θ* is the fractional change in the population parameter for an individual with a covariate value different from the median value (Eq. 2) or a reference value (Eq. 4). *k* stands for the exponential scaling factor and in case of body weight, it was either estimated or fixed to a certain value (0.75 for clearance and 1 for volume parameters) [38].

A randomization test was performed to calculate the probability of identifying falsely significant covariates [39]. A total of 200 new datasets were generated by shuffling 200 times the sequence of the covariate values between individuals in the randomization column. A base model (without a parameter–covariate relationship) was fitted to the original dataset. Then, a full model (with a parameter–covariate relationship) was fitted to the original and the randomized datasets, and the ΔOFVs from the base model were computed. Based on the distribution of the difference in OFV between the two models for each of the 200 datasets, the actual drop in OFV required to reach the 5 % significance level was calculated.

Data analysis was performed in two separate steps. As a first step, only total bevacizumab concentration–time (PK) data were included in the analysis and a population PK model for bevacizumab was developed to gain information on the PK profile of the drug in the current study population. A previously published model by Lu et al. [26], which describes bevacizumab data in a similar study population in terms of demographic and study characteristics, was used as a reference to evaluate whether the present PK data are in reasonable agreement with the previous observations. In the second step of data analysis, a simultaneous fit of total bevacizumab and free VEGF_165_ concentration–time data was performed to develop a binding model for bevacizumab using a TMDD modeling approach. The simultaneous data analysis allowed for the exploration of the influence of VEGF_165_ on bevacizumab disposition and eliminated the effect of potential shrinkage in PK parameters on the estimation of VEGF-related parameters.

### PK model

Total bevacizumab concentrations in serum were expressed in mg/L.

In the first step of data analysis, one- and two-compartment models with first-order elimination were investigated to describe bevacizumab concentration changes over time.

The effect of body weight, age and gender was tested on all PK parameters taking into account prior knowledge on the highly influential covariates [25, 26] and the clinical relevance of the available covariates. In addition, the influence of VEGF-A SNPs (−2578C/A, −1154G/A and −634G/C) was only explored on clearance parameters, as these polymorphisms are associated with VEGF production or promoter activity [12–14].

Inter-individual variability was investigated in all model parameters, and an error model was developed to describe the unexplained residual variability.

All parameters of the developed PK model were estimated, and the PK parameter estimates reported by Lu et al. [26] were only used as initial estimates. The ability of the proposed model to yield plausible parameter estimates was tested by comparing them with the parameter estimates obtained by the model of Lu et al., which predicts the concentration–time profile of bevacizumab in a larger study population.

### TMDD (binding) model

Total bevacizumab and free VEGF_165_ concentrations in serum were expressed in nM. Modeling was performed on log-transformed data.

In the second step of data analysis, the structural and covariate form of the developed PK model for bevacizumab was linked with a QSS TMDD model to simultaneously describe total bevacizumab and free VEGF_165_ concentration–time profiles.

The QSS equations [22–24] for the concentrations of total bevacizumab (*C*_tot_), total VEGF_165_ (*R*_tot_), free bevacizumab (*C*), bevacizumab–VEGF_165_ complex (*RC*) and free VEGF_165_ (*R*) are shown below:

5 $$\frac{{{\text{d}}C_{\text{tot}} }}{{{\text{d}}t}} = \frac{{{\text{In}}\left( t \right)}}{{V_{1} }} - \left( {\frac{\text{CL}}{{V_{1} }} + \frac{Q}{{V_{1} }}} \right) \cdot C - \frac{{R_{\text{tot}} \frac{{{\text{CL}}_{RC} }}{{V_{1} }} C}}{{K_{\text{ss}} + C}} + \frac{{\frac{Q}{{V_{2} }}A_{2} }}{{V_{1} }}$$

6 $$\frac{{{\text{d}}R_{\text{tot}} }}{{{\text{d}}t}} = k_{\text{in}} - k_{\text{out}} \cdot R_{\text{tot}} - \left( {\frac{{{\text{CL}}_{RC} }}{{V_{1} }} - k_{\text{out}} } \right)\frac{{R_{\text{tot}} \cdot C}}{{K_{\text{ss}} + C}}$$

7 $$C = \frac{1}{2}\left[ {\left( {C_{\text{tot}} - R_{\text{tot}} - K_{\text{ss}} } \right) + \sqrt {\left( {C_{\text{tot}} - R_{\text{tot}} - K_{\text{ss}} } \right)^2 + 4 \cdot K_{\text{ss}} \cdot C_{\text{tot}} } } \right]$$

8 $$RC = \frac{{R_{\text{tot}} \cdot C}}{{K_{\text{ss}} + C}}$$

9 $$R = R_{\text{tot}} - RC$$

In these equations, In(t) is the infusion rate; *A*_2_ is the amount of the free bevacizumab in the peripheral compartment; *V*_1_ and *V*_2_ are the volumes of distribution of the central and peripheral compartment, respectively; CL is the elimination clearance of free bevacizumab; CL_*RC*_ is the elimination clearance of the bevacizumab–VEGF_165_ complex; *Q* is the intercompartmental clearance. *k*_in_ and *k*_out_ are the production (zero-order) and elimination (first-order) rate constants of free VEGF_165_. *K*_ss_ is the steady-state constant that defines the QSS among free bevacizumab, free VEGF_165_ and bevacizumab–VEGF_165_ complex and is described by the following equation:

10 $$K_{\text{ss}} = \frac{{k_{\text{int}} + k_{\text{off}} }}{{k_{\text{on}} }}$$

*k*_int_, *k*_off_ and *k*_on_ are the elimination (first-order), dissociation (first-order) and binding (second-order) rate constants of the bevacizumab–VEGF_165_ complex.

VEGF-A SNPs (−2578C/A, −1154G/A and −634G/C) were only tested for significance on VEGF-related parameters.

Inter-individual variability was investigated in all PK and VEGF-related parameters, and an error model was developed to describe the unexplained residual variability.

All parameters of the developed TMDD model were estimated, and the population PK parameter estimates from the first step of data analysis were only used as initial estimates.

### Model evaluation

The predictive performance of the developed models for bevacizumab (PK and TMDD model) was evaluated using prediction-corrected visual predictive checks (pcVPCs) [40, 41]. A total of 1000 simulated datasets were generated, and the 95 % confidence intervals for the median, 10th and 90th percentiles of the simulated data were compared to the median, 10th and 90th percentiles of the observed data. The uncertainty in model parameters was expressed by the relative standard error (RSE) obtained by the covariance step in NONMEM.

## Results

### Patients and samples

The current analysis was based on 86 total bevacizumab and 93 free VEGF_165_ serum concentrations collected from 19 adult patients with stage IV CRC. None of the measured concentrations was below the limit of quantification. Blood samples (median 4, 2–10 per patient) were collected before bevacizumab administration and immediately after the end of infusion.

Ten patients received bevacizumab in combination with FOLFIRI or FOLFOX treatment, whereas nine patients were administered bevacizumab together with CAPIRI treatment. Only three patients switched over to bevacizumab monotherapy (one patient from each treatment group). The patient on BEV–CAPIRI treatment had received nine cycles of bevacizumab before changing to bevacizumab monotherapy.

The study population was predominately male (58 %), with a median age of 60 years and median body weight of 70 kg. The most frequent genotypes were VEGF-2578CA (58 %) for VEGF-2578C/A, VEGF-1154GG (53 %) for VEGF-1154G/A and VEGF-634GG (74 %) for VEGF-634G/C SNPs.

A summary of the patient and study characteristics is shown in Table 1.

**Table 1:** Patient and study characteristics

Characteristic	Value
Dose (mg/kg)	5 or 10 biw, 7.5 tiw
Duration of infusion (min)	85 (40–90)
Treatment period (days)	
BEV–FOLFIRI or BEV–FOLFOX treatment	127.5 (27–348)
BEV–CAPIRI treatment	174 (71–251)
Actual body weight (kg)	70 (50–94)
Age (years)	60 (37–73)
Gender (M/F)	11/8 (58/42 %)
Total bevacizumab concentrations (mg/L)	
Pre-dose concentrations	83.6 (13.5–172)
Post-dose concentrations	203.8 (71.1–412)
Free VEGF_165_ concentrations (ng/L)	
Pre-dose concentrations	187.7 (52.3–451.6)
Post-dose concentrations	76.5 (16.9–371.6)
Bevacizumab samples/cycle	
BEV–FOLFIRI or BEV–FOLFOX treatment	2 (1–8)
BEV–CAPIRI treatment	4 (1–10)
VEGF_165_ samples/cycle	
BEV–FOLFIRI or BEV–FOLFOX treatment	2 (1–8)
BEV–CAPIRI treatment	3.5 (1–9)
VEGF_2578C/A_ (CC^a^/AA^b^/CA^c^)	2/6/11 (10/32/58 %)
VEGF_1154G/A_ (GG^a^/AA^b^/GA^c^)	10/2/7 (53/10/37 %)
VEGF_634G/C_ (GG^a^/CC^b^/GC^c^)	14/1/4 (74/5/21 %)

Values are either expressed as median (range) or absolute values (%)

*biw* every 2 weeks, *tiw* every 3 weeks, *M* male, *F* female

^a^Wild type; ^b ^Homozygous; ^c ^Heterozygous

### VEGF assay

The ability of the Quantikine^®^ human VEGF ELISA assay [32] to detect free or total VEGF_165_ in the presence of bevacizumab was tested in vitro by mixing VEGF_165_ standards (1000 and 250 ng/L) with increasing bevacizumab concentrations. The measured VEGF_165_ concentrations were equal to zero when bevacizumab was in excess confirming that only free VEGF_165_ can be detected by this VEGF ELISA assay, otherwise the measured VEGF_165_ concentrations should be identical to the concentrations of VEGF_165_ standards (1000 and 250 ng/L). Mean (RSE %) VEGF_165_ concentrations for VEGF_165_ to bevacizumab 1:0, 1:0.1, 1:1 and 1:1000 molar ratios are shown in Table 2.

**Table 2:** In vitro VEGF_165_ concentrations after addition of bevacizumab

VEGF_165_ standards (ng/L)	VEGF_165:_bevacizumab molar ratio	VEGF_165_ concentrations (ng/L)
1000	1:0	1120.2 (2.67)
	1:0.1	448.5 (1.66)
	1:1	452.2 (0.83)
	1:1000	0 (0)
250	1:0	172.4 (4.33)
	1:0.1	79.1 (4.72)
	1:1	79.1 (4.72)
	1:1000	0 (0)

VEGF_165_ concentrations are expressed as average (RSE %)

### PK model

A two-compartment model with first-order elimination was found to describe bevacizumab concentration changes over time better than a one-compartment model (ΔOFV = −18.9). The selection of the final structural model was further supported by basic goodness-of-fit plots and a previously published model for bevacizumab [26]. Goodness-of-fit plots showed no apparent bias in residuals over time or across population-predicted bevacizumab concentrations (Supplemental Fig. 2).

*V*_1_ and *V*_2_ were found to be 3.14 and 2.63 L, respectively. Bevacizumab CL was 0.17 L/day, and *Q* was 0.36 L/day.

Inter-individual variability could be identified for CL (23 %) and *V*_1_ (15 %). The residual variability was explained by a proportional error model (24 %).

In the stepwise covariate analysis, the effect of several covariates (body weight, age, gender and VEGF-A SNPs) was explored on the PK parameters. None of the available covariates provided an increase of 6.64 points in OFV for backward deletion (*p* < 0.01, for one parameter difference). Body weight was incorporated with an allometric function in all clearance and volume parameters to ensure model stability considering the strong biological covariate relationship [38, 42] and prior information from studies in patients with solid tumors receiving bevacizumab [25, 26].

Model parameters could be estimated with acceptable precision (RSE < 25 %) except for *V*_*2*_ (RSE 50 %), *Q* (RSE 146 %) and inter-individual variability in *V*_1_ (RSE 38 %). The parameter estimates from the PK model and their RSEs are represented in Table 3.

**Table 3:** Population parameter estimates from the PK model (bevacizumab analyzed alone) and TMDD model (bevacizumab and VEGF_165_ analyzed simultaneously)

Parameter	PK model		TMDD model	
	Typical value (RSE %)	IIV, CV % (RSE %)	Typical value (RSE %)	IIV, CV % (RSE %)
CL (L/day)	0.17 (11)	23 (23)	0.18 (6)	20 (25)
*V*_1_ (L)	3.14 (7)	15 (38)	3.23 (7)	22 (36)
*Q* (L/day)	0.36 (146)		1.38 (19)	
*V*_2_ (L)	2.63 (50)		3.1 (18)	
BM_0_ (ng/L)			212 (8)	33 (23)
*k*_out_ (/day)			0.401 (14)	
*K*_ss_ (nM)			267 (22)	
Prop. error_bev_ (%)	24 (13)		28 (15)	
Prop. error_VEGF165_ (%)			32 (14)	

*CL* clearance, *V*_*1*_ volume of central compartment, *Q* intercompartmental clearance, *V*_*2*_ volume of peripheral compartment, *BM*_*0*_ baseline VEGF_165_, *k*_*out*_ elimination rate constant of VEGF_165_, *K*_*ss*_ steady-state constant, *RSE* relative standard error, *IIV* inter-individual variability, *CV* coefficient of variation, *bev* bevacizumab

### TMDD (binding) model

**Fig. 1:**
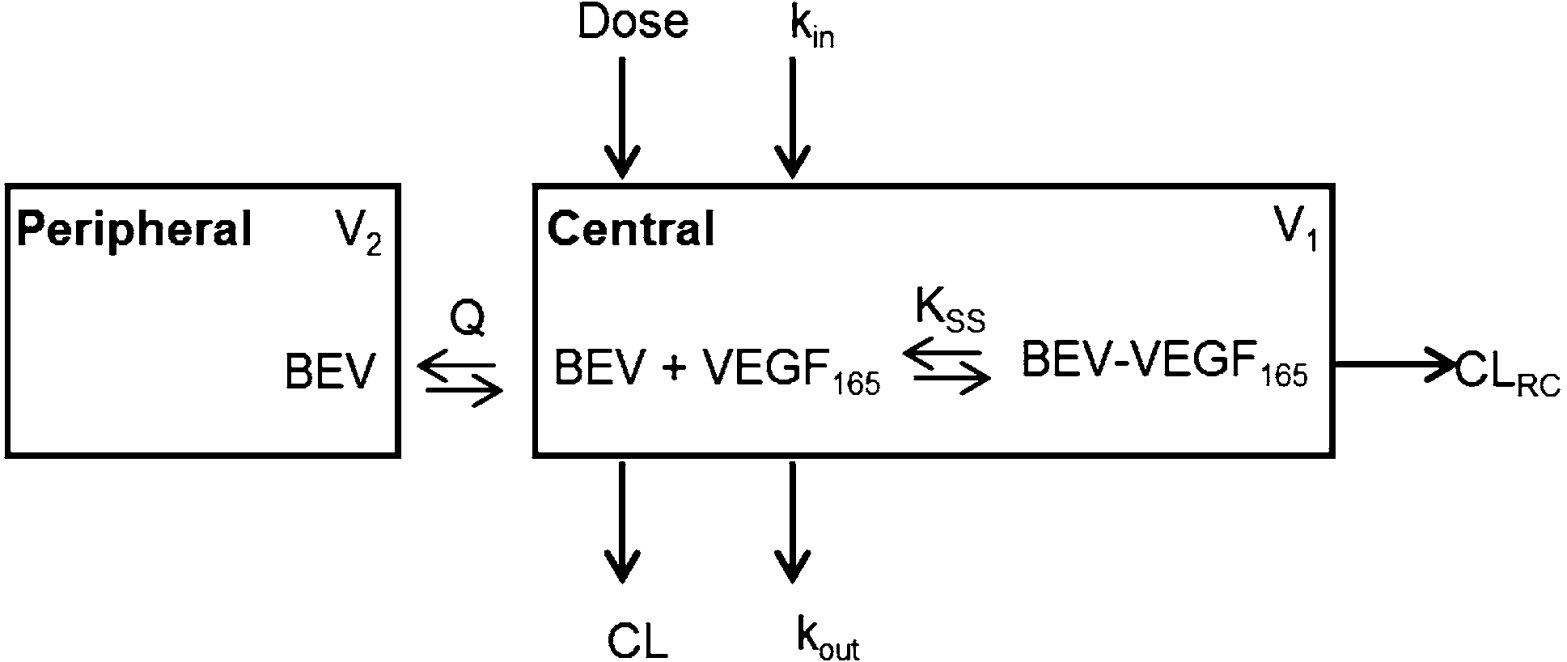
Structure of the binding model for bevacizumab–VEGF_165_ interaction. The approximation CL_*RC*_ = CL was used for purposes of model fitting

A QSS TMDD model was applied to explore the in vivo interaction between bevacizumab and its soluble target, VEGF_165_. In this model, free bevacizumab is eliminated from the central compartment through two pathways: (1) degradation (linear CL) and (2) binding to VEGF_165_ and subsequent degradation of the bevacizumab–VEGF_165_ complex (linear CL_*RC*_). The structure of the binding model is shown in Fig. 1.

The developed model adequately described the time course of total bevacizumab and free VEGF_165_ serum concentrations in the current study population. The estimated CL of free bevacizumab was 0.18 L/day, and *Q* was 1.38 L/day. The information in the present data was not sufficient to allow for a separate estimation of the elimination clearance of the bevacizumab–VEGF_165_ complex, which was therefore set equal to the CL of the free bevacizumab (CL_*RC*_ = CL). Baseline VEGF_165_ (free VEGF_165_ at time 0, BM_0_) was 0.0053 nM corresponding to 212 ng/L (assuming a 1:1 molecular interaction), and *k*_out_ was 0.401 day^−1^. *K*_ss_ was found to be 267 nM. *k*_in_, which is defined as the typical value of BM_0_ times the typical value of *k*_out_ (BM_0,*P*_ × *k*_out,*P*_), was 85 ng/L/day.

Inter-individual variability was assigned to the following parameters: CL, *V*_1_ and BM_0_. The residual variability in total bevacizumab and free VEGF_165_ concentrations was explained by an additive error model on log-transformed data.

No direct relationship between VEGF-A SNPs and BM_0_, *k*_out_ or *K*_ss_ was identified. However, patients with VEGF-2578AA (homozygous), VEGF-634CC (homozygous) and VEGF-634GC (heterozygous) genotypes seem to have a larger *K*_ss_. The effect of VEGF-634G/C polymorphism on *K*_ss_ becomes significant (ΔOFV = −8.6) only after the inclusion of VEGF-2578C/A polymorphism in *K*_ss_ (ΔOFV = −6). A plot of ΔOFV versus individuals indicated that one specific individual was driving the relationship between VEGF-2578C/A and *K*_ss_ (data not shown). A randomization test was then performed to explore whether the inclusion of these covariate relationships was falsely significant. It was concluded that the observed trends between VEGF-2578C/A and *K*_ss_ or VEGF-634G/C and *K*_ss_ did not reach statistical significance as the required drop in OFV after the inclusion of VEGF-2578C/A or VEGF-634G/C was 6.8 points or 6.7 points, respectively (*p* < 0.05, forward inclusion of one parameter).

All the PK and VEGF-related parameter estimates from the binding model and their RSEs are represented in Table 3. The RSEs of the fixed and random effects remained well below 25 % except for the inter-individual variability in *V*_1_ (RSE 36 %), indicating that the parameters could be estimated with acceptable precision. A low ε-shrinkage (10 %) suggests that the model diagnostics are reliable. However, η-shrinkage was relatively high for *V*_1_ (34 %) but <22 % for CL and BM_0_.

### Evaluation of the TMDD (binding) model

**Fig. 2:**
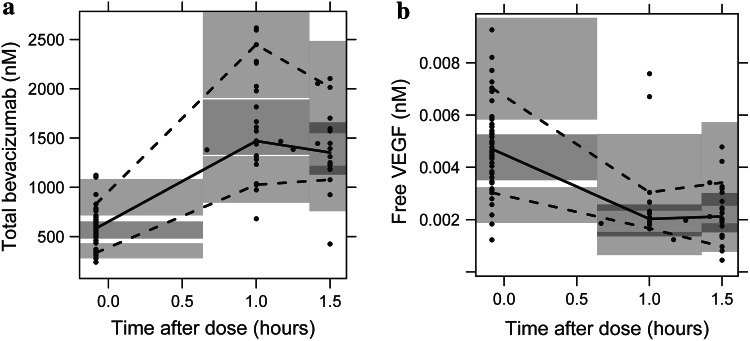
Prediction-corrected visual predictive checks of the binding model based on 1000 simulations (panel **a** total bevacizumab, panel **b** free VEGF_165_). Median (*solid line*), 10th and 90th percentiles (*dashed lines*) of the observed data (*circles*) are compared to the 95 % confidence intervals (*shaded areas*) for the median, 10th and 90th percentiles of the simulated data

The TMDD model had a good predictive performance, as expressed by the results of the VPC depicted in Fig. 2. It can adequately describe the time course of total bevacizumab and free VEGF_165_ concentrations as well as the observed variability in the study population.

### Simulations

**Fig. 3:**
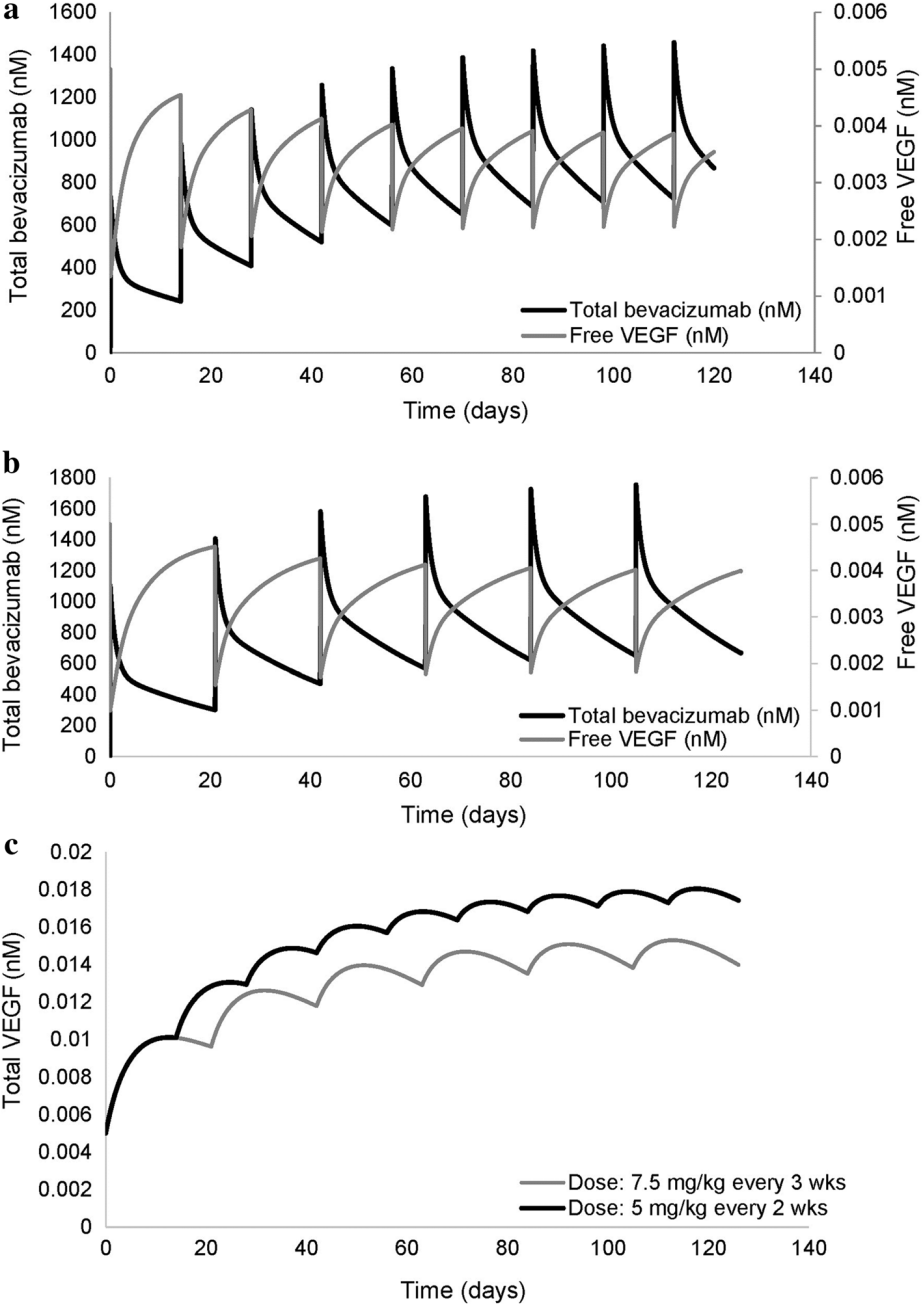
Model-predicted concentrations of total bevacizumab, total and free VEGF_165_ for a typical patient of 70 kg. Panels **a**, **b** show the total bevacizumab and free VEGF_165_ concentration profiles at doses of 5 and 7.5 mg/kg, respectively. Panel **c** depicts the total VEGF_165_ profiles over time for the two dosing regimens

The concentration–time profiles of total bevacizumab, total and free VEGF_165_ for a typical patient of 70 kg receiving bevacizumab either at a dose of 5 mg/kg every 2 weeks or 7.5 mg/kg every 3 weeks (the most frequent dosing regimens in the study population) are shown in Fig. 3.

A significant drop in the free VEGF_165_ levels was observed upon administration of the first dose (73 % for the lower dose and 80 % for the higher dose) followed by a less pronounced decline on subsequent doses attributed to the reversible formation and accumulation of bevacizumab–VEGF_165_ complexes (Fig. 3a, b, respectively). The total VEGF_165_ concentrations increased over time, in both dosing regimens, up to a level where no more complexes could be formed. The extent of total VEGF_165_ accumulation seemed less at the higher dose compared to the lower dose, while the decline in free VEGF_165_ levels was slightly increased (54 % compared to 42 % for the lower dose, Fig. 3c).

## Discussion

In this study, a binding model for bevacizumab was developed to characterize its PK behavior, to describe its binding properties to VEGF_165_ and to assess the influence of relevant covariates (e.g., genetics) on the relationship between bevacizumab and VEGF_165_ in adult patients with stage IV CRC. Sparse bevacizumab and VEGF_165_ data were collected during routine clinical practice from 19 adult patients following bevacizumab treatment in combination with chemotherapy (FOLFIRI, FOLFOX or CAPIRI). Data analysis was performed in two distinct steps using nonlinear mixed-effects modeling.

In the first step of data analysis, a PK model for bevacizumab was developed and the model-predicted profiles were compared to the profiles reported by Lu et al. [26] to evaluate whether the data collected in the current study were in accordance with previous observations. Although the two-compartment model did not provide a pronounced improvement of the model fit over a one-compartment model, it was found to support the previously reported model structure and parameters and was therefore selected.

In the second step of data analysis, a TMDD model using the QSS approximation [22–24] was developed to characterize the in vivo bevacizumab–VEGF_165_ interaction based on a simultaneous analysis of total bevacizumab and free VEGF_165_ serum concentrations. The simultaneous data fitting allowed for the evaluation of the effect of bevacizumab on the reduction in free VEGF_165_ levels and shed light on the in vivo binding affinity of bevacizumab to VEGF_165_ as most of the current data come from in vitro biological studies [43, 44]. Furthermore, it provided some valuable information on the PK properties of both bevacizumab and VEGF_165_ in patients with cancer.

The TMDD model could adequately describe total bevacizumab and free VEGF_165_ profiles observed during bevacizumab treatment. The simultaneous data analysis allowed for a higher precision in the estimation of PK parameters compared to the analysis of bevacizumab data alone, indicating that VEGF_165_ concentrations may carry information on bevacizumab disposition. In this model, the typical volume of the central and peripheral compartment was similar to the values previously reported by Lu et al. [26] and Gaudreault et al. [45]. Bevacizumab clearance was found to be consistent with that observed by Lu et al. (0.21 L/day) [26] and Gordon et al. (0.19–0.36 L/day for a typical patient of 70 kg) [46]. The estimated free VEGF_165_ levels at baseline as well as the elimination rate constant, *k*_out_, of free VEGF_165_ were also in line with published values in patients with advanced cancer [47, 48]. However, a much larger *K*_ss_ (267 nM) than the in vitro equilibrium dissociation constant (KD) of bevacizumab for VEGF-A (1.8–20 nM) [1, 44] was observed. This finding is in agreement with the QSS approximation that often predicts a greater *K*_ss_ value in vivo than the in vitro KD [21, 23].

Model-based simulations (Fig. 3) revealed a significant drop (73–80 %) in the free serum VEGF_165_ concentrations upon administration of the first dose of bevacizumab, which reaches a pseudo-steady-state after multiple doses. A similar behavior was suggested by Stefanini et al. [49] in case bevacizumab is confined to the blood compartment. They mentioned a decline of 87 % in free serum VEGF_165_ levels after the first dose of bevacizumab. Model predictions regarding the effect of bevacizumab on the reduction in free VEGF_165_ levels in serum are of particular importance as they could indicate when dose adjustments are needed to achieve sufficient VEGF-A blockade and thus optimal anti-angiogenic activity of bevacizumab. Increases in total serum VEGF_165_ concentrations were also noticed, as in previous studies by Gordon et al. [46] and Stefanini et al. [49]. This increase in total VEGF_165_ could be a result of bevacizumab–VEGF_165_ complex dissociation, a decrease in VEGF_165_ clearance caused by the complexation process [50] or a constant production rate of VEGF_165_.

The effect of demographic characteristics and genetics on the PK and VEGF-related parameters in the target population was explored. Among all covariates tested, none was found to be significant according to the predefined statistical criteria. The difficulty in identifying any significant covariate effects could be attributed to the sparse sampling schedule and the small size of the study population. Thus, the developed TMDD (binding) model did not include any covariates except for body weight in all clearance and volume parameters to ensure model stability, considering the strong biological prior [38, 42] and the outcome of previous analyses [25, 26]. Despite the reported gender differences in bevacizumab clearance and volume of distribution [26, 45], the gender effect was not retained in the final model because gender and patient body weight are often strongly correlated, and thus, the inclusion of body weight is usually sufficient to completely describe the gender influence. The effect of serum albumin and alkaline phospatase on clearance could not be assessed in the current study since data were not available for all patients. The influence of co-administered chemotherapy (FOLFIRI, FOLFOX, CAPIRI) was not tested on bevacizumab PK, as more information would be needed to unravel potential drug–drug interactions. Nevertheless, published data indicate no significant PK interactions between bevacizumab and other antineoplastic agents [1, 26].

Interestingly, a larger *K*_ss_ value was observed for patients with VEGF-2578AA, VEGF-634CC and VEGF-634GC genotypes. It is noteworthy that when the covariate effect of VEGF-2578C/A or VEGF-634G/C polymorphism was added in *K*_ss_, none of the model-predicted parameters was substantially affected except for the value of *K*_ss_, which was reduced from 267 to 221 or 176 nM, respectively. This finding indicates that VEGF-2578C/A and VEGF-634G/C polymorphisms might be predictive for the binding affinity of bevacizumab to VEGF_165_, as *K*_ss_ and affinity of the drug to its molecular target are correlated (Eq. 10). Larger cohort studies in bevacizumab-treated patients are needed though, to confirm these results and to assess the potential role of VEGF-2578C/A and VEGF-634G/C polymorphisms in guiding patient and dose selection.

Some limitations of our study should be also addressed. The elimination clearance of the bevacizumab–VEGF_165_ complex could not be estimated and it was set equal to the clearance of the free bevacizumab, allowing for a simultaneous model fit of total bevacizumab and free VEGF_165_ serum concentrations. Free bevacizumab (~150 kDa) is known to undergo proteolytic catabolism mediated by the neonatal Fc receptor (FcRn), which contributes to the slow elimination of the drug from the systemic circulation [12]. It is likely that the bevacizumab–VEGF_165_ complex (assuming a 1:1 molecular interaction), which has a similar molecular weight with bevacizumab (~190 kDa) and is salvaged from degradation through binding of its Fc moiety to FcRn, exhibits a similar elimination rate. This hypothesis is further supported by Hsei et al. [50], who observed no statistically significant difference between the clearance of the free bevacizumab and the bevacizumab–VEGF_165_ complex in rats. In the current study, the free drug and the complex were also assumed to be distributed in the same space, given that such large molecules would extravasate relatively slow. However, Hsei et al. [50] demonstrated a small, though significant change (20–25 %) in the volumes of distribution, implying that free bevacizumab and bevacizumab–VEGF_165_ complex might not be distributed exactly in the same space. A future study in humans, which would provide information on the concentration–time profile of the bevacizumab–VEGF_165_ complex, could therefore shed more light on the disposition properties of the complex and detect potential interspecies differences. Moreover, it was hypothesized that only one type of complex can be formed through monomeric binding (bevacizumab bound to one molecule of VEGF_165_). The possibility of formation of other complexes through multimeric binding (e.g., bevacizumab bound to two molecules of VEGF_165_, VEGF_165_ bound to two molecules of bevacizumab or other immune complexes) was ignored, as the bevacizumab bioassay could only detect the complex formed between bevacizumab and one molecule of VEGF_165_. The quantification of higher-order immune complexes is considered extremely difficult due to their rapid elimination from the systemic circulation [51]. Their assessment, though, could be proved valuable in reflecting an additional clearance pathway for bevacizumab. The effect of platelets on taking up VEGF and bevacizumab [52] was not included in the current model but it would be useful to be added when more data become available, as it could provide information on the fluctuation of serum VEGF levels during bevacizumab treatment.

## Conclusion

In conclusion, the developed binding model adequately characterizes the PK of bevacizumab and the relationship between bevacizumab and VEGF_165_ in adult patients with stage IV CRC receiving bevacizumab treatment in combination with chemotherapy. To the best of our knowledge, this is the first time a TMDD model was applied to characterize the in vivo interaction of bevacizumab with its soluble ligand, VEGF_165_. Although no significant effect of VEGF-A polymorphisms on the relationship between bevacizumab and VEGF_165_ was identified, correlations between the binding affinity of bevacizumab to VEGF_165_ and the VEGF-2578C/A and VEGF-634G/C SNPs were noticed. This model could serve as a basis for further studies to elucidate the role of VEGF-A polymorphisms and serum VEGF levels in treatment response and to support dose rationale for bevacizumab when combined with chemotherapy.

## Electronic supplementary material

Below is the link to the electronic supplementary material.

Supplementary material 1 (DOCX 444 kb)
