# Supplementary material for: A pharmacokinetic binding model for bevacizumab and VEGF165 in colorectal cancer patients
Source: Cancer Chemother Pharmacol. 2015 Feb 17;75(4):791–803. doi: 10.1007/s00280-015-2701-3 (PMC4365273; doi:10.1007/s00280-015-2701-3)
Supplement: Supplementary file 1 — Supplementary material 1 (DOCX 444 kb) [file 280_2015_2701_MOESM1_ESM.docx]

**A pharmacokinetic binding model for bevacizumab and VEGF_165_ in colorectal cancer patients**

**Cancer Chemotherapy and Pharmacology**

Eirini Panoilia^1^, Emilie Schindler^2^, Epaminontas Samantas^3^, Gerasimos Aravantinos^4^, Haralabos P. Kalofonos^5^, Christos Christodoulou^6^, George P. Patrinos^1^, Lena E. Friberg^2^, Gregory Sivolapenko^1^

^1^Department of Pharmacy, University of Patras, Patras, Greece; ^2^Department of Pharmaceutical Biosciences, Uppsala University, Uppsala, Sweden; ^3^3^rd^ Department of Medical Oncology, “Agii Anargiri” Cancer Hospital, Kalyftaki Nea Kifissia, Greece; ^4^2^nd^ Department of Medical Oncology, “Agii Anargiri” Cancer Hospital, Kalyftaki Nea Kifissia, Greece; ^5^Division of Medical Oncology, University Hospital of Patras, Rion, Greece; ^6^2^nd^ Department of Medical Oncology, “Metropolitan” Hospital, Athens, Greece

Corresponding author: E. Panoilia, Pharmacokinetics Laboratory, Department of Pharmacy, University of Patras, 26504 Patras, Greece, e-mail: [eipanoilia@upatras.gr](mailto:eipanoilia@upatras.gr)

**Supplemental Fig. 1** Sample collection schedule for patients on BEV-FOLFIRI, BEV-FOLFOX and BEV-CAPIRI treatment

Panel a shows the pre- and post-dose blood samples drawn from patients on BEV-FOLFIRI or BEV-FOLFOX treatment (both treatments are administered in 2-week cycles). Panel b depicts the pre- and post-dose blood samples collected from patients on BEV-CAPIRI treatment (treatment is administered in 3-week cycles). Bevacizumab infusion is indicated by the white arrow. The two blood samples (pre- and post-dose), which are intended for total bevacizumab and free VEGF_165_ measurements, are collected at the cycles indicated by the black arrows. The pre-dose sample, which is only intended for free VEGF_165_ measurement, is collected at the cycle indicated by the grey arrow.

**Supplemental Fig. 2** Residual goodness-of-fit plots for the PK model (bevacizumab analyzed alone)

The residual plots of serum total bevacizumab concentrations for the final PK model. Conditional weighted residuals versus time (a), conditional weighted residuals versus population-predicted bevacizumab concentrations (b). Blue points are individual data. Black solid lines represent the unit line at zero. Black dashed lines represent the absolute value of conditional weighted residuals of two. Red solid lines represent the LOESS smoothed lines.

**Supplemental Fig. 1**

a

C1 C2 C3 C4 C5 C6 C7 C8 C9 C10 C11 C12 C13 C14 C15 C16 C17 C18 C19 C20 C21 C22 C23 C24

Cycles

Prior to and after the end of infusion

Prior to and after the end of infusion

Prior to and after the end of infusion

Prior to and after the end of infusion

Prior to and after the end of infusion

Prior to infusion

Prior to and after the end of infusion

b

C1 C2 C3 C4 C5 C6 C7 C8 C9 C10 C11 C12 C13 C14

Cycles

Prior to and after the end of infusion

Prior to and after the end of infusion

Prior to and after the end of infusion

Prior to and after the end of infusion

Prior to infusion

Prior to and after the end of infusion

**Supplemental Fig. 2**

| a | b |
| --- | --- |
| 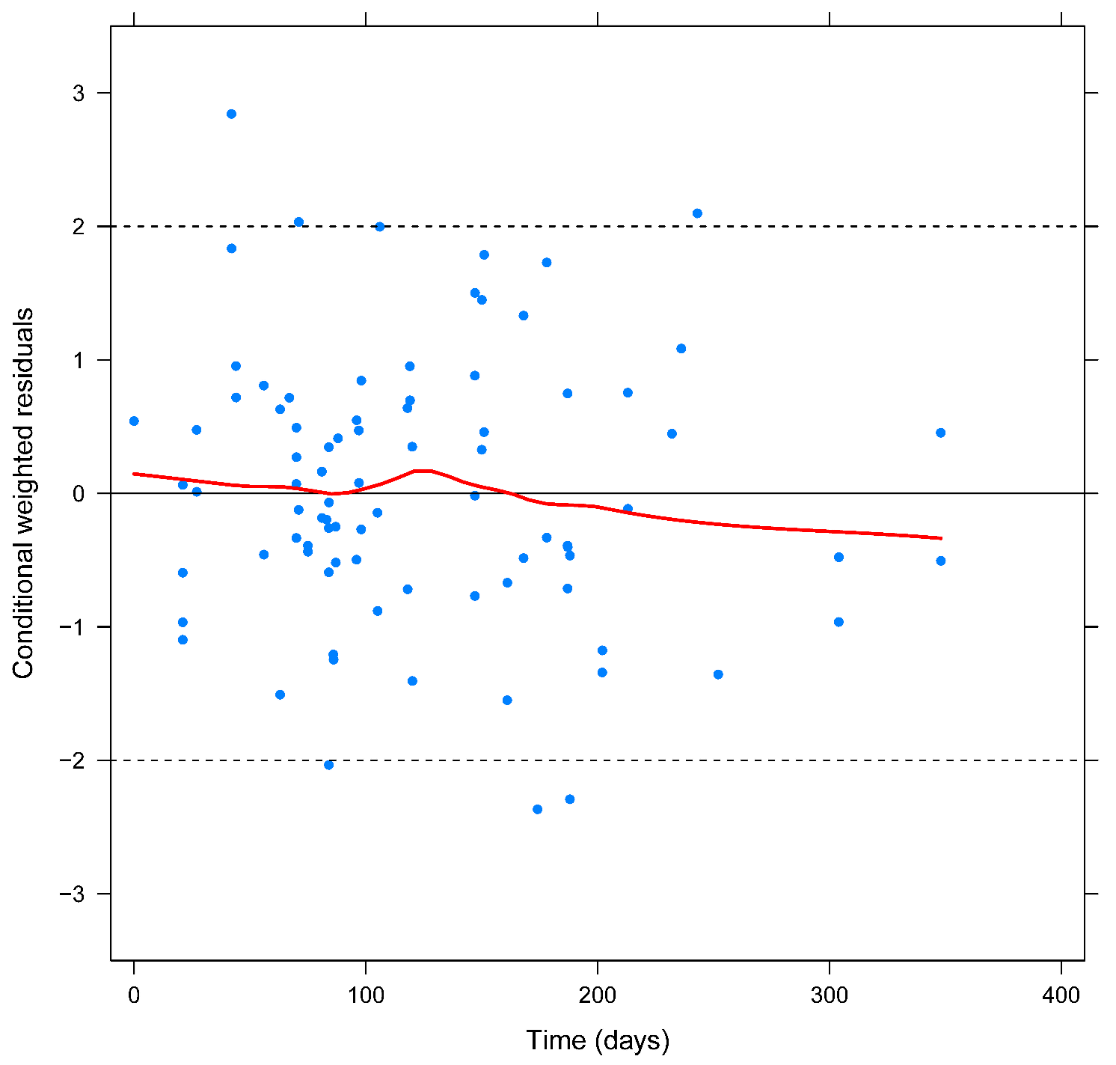 | **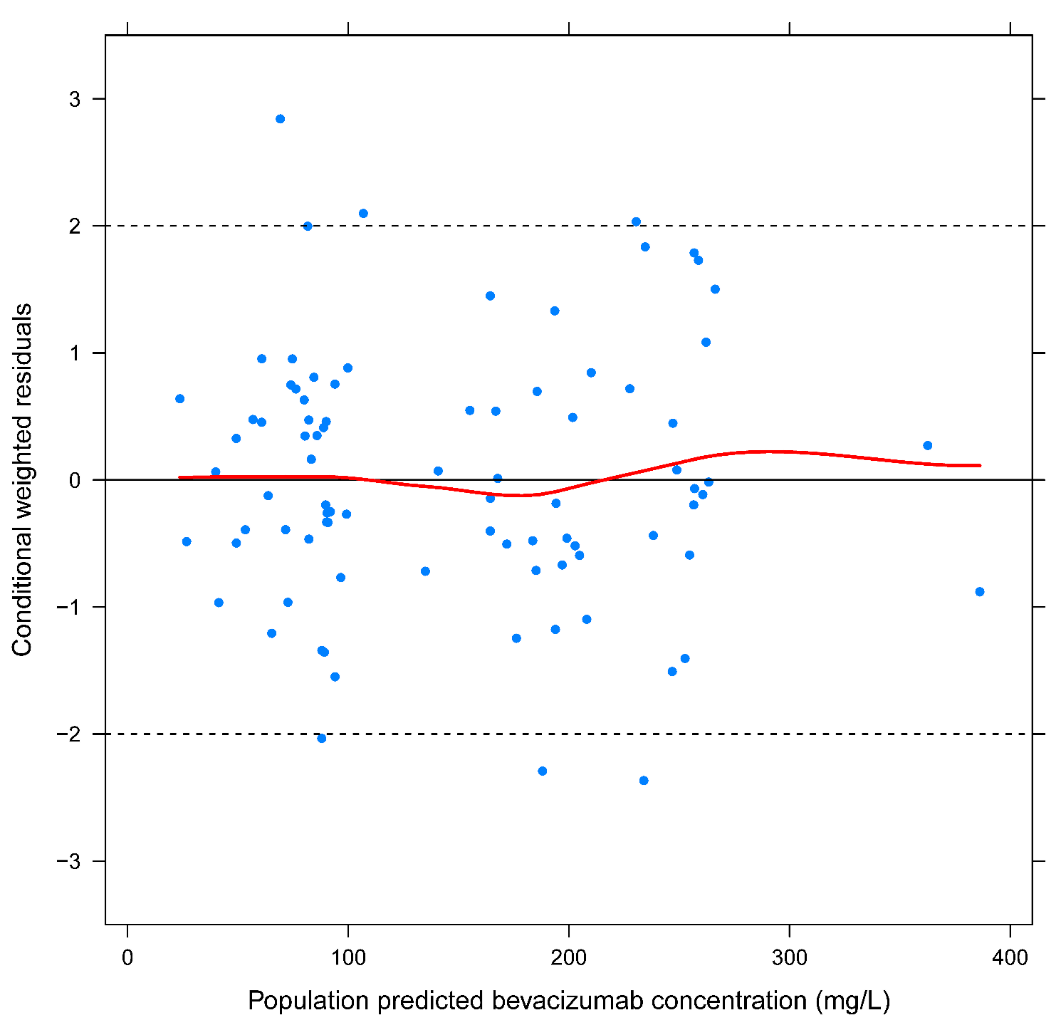** |
